# Explanations and Causal Judgments Are Differentially Sensitive to Covariation and Mechanism Information

**DOI:** 10.3389/fpsyg.2022.911177

**Published:** 2022-08-01

**Authors:** Ny Vasil, Tania Lombrozo

**Affiliations:** ^1^Department of Psychology, California State University, East Bay, Hayward, CA, United States; ^2^Concepts and Cognition Lab, Department of Psychology, University of California Berkeley, Berkeley, CA, United States; ^3^Department of Psychology, Princeton University, Princeton, NJ, United States

**Keywords:** explanation, causation, mechanism, covariation, generalization

## Abstract

Are causal explanations (e.g., “she switched careers because of the COVID pandemic”) treated differently from the corresponding claims that one factor caused another (e.g., “the COVID pandemic caused her to switch careers”)? We examined whether explanatory and causal claims diverge in their responsiveness to two different types of information: covariation strength and mechanism information. We report five experiments with 1,730 participants total, showing that compared to judgments of causal strength, explanatory judgments tend to be *more* sensitive to mechanism and *less* sensitive to covariation – even though explanatory judgments respond to both types of information. We also report exploratory comparisons to judgments of understanding, and discuss implications of our findings for theories of explanation, understanding, and causal attribution. These findings shed light on the potentially unique role of explanation in cognition.

## Introduction

In a well-known episode from 19th century medicine, Ignaz Semmelweis puzzled over a correlation between the clinic in which a woman gave birth (the First Clinic vs. the Second Clinic of the Vienna General Hospital), and her probability of succumbing to Puerperal Fever after the birth (10% vs. less than 4%). Expectant mothers (among others) seemed to accept that there was some causal relationship between giving birth in the First Clinic and the increased maternal mortality – indeed, women begged to be admitted to the Second Clinic, and Semmelweis entertained a variety of hypotheses about the relevant causal factor. Although the evidence for a causal relationship was reasonably strong, what seemed to be missing was an explanation: *why* were women who gave birth in the First Clinic at greater risk?

This example illustrates a way in which causal and explanatory judgments potentially come apart. There may be situations in which we feel compelled to believe that a causal relationship exists (based on a strong pattern of correlations and/or evidence that manipulating C produces changes in E), and we might agree that “C is a cause of E.” And yet, we might find the corresponding explanation, “E occurred because C,” unsatisfying. What is missing to support the explanation? One important factor, we contend, is knowledge of a plausible *causal mechanism*. This is precisely what Semmelweis pursued: he went on to test a variety of potential mechanisms, and discovered that “cadaveric matter” was being transported on the hands of doctors who both performed autopsies and delivered babies in the First Clinic, but not by the midwives who delivered babies in the Second Clinic. When he instated an intervention that required hand-washing, the instances of Puerperal Fever decreased. In more contemporary terms, we might explain the correlation between birth clinic and maternal mortality in terms of the germs (group A Streptococcus bacteria) introduced into each clinic at different rates (Ataman et al., 2013; Anderson, 2014).

In this article we investigate whether causal mechanism information and statistical evidence (such as the covariation noted by Semmelweis) play different roles in judgments concerning explanatory vs. causal relationships. Although we might expect both causal and explanatory judgments to be influenced by knowledge of a plausible mechanism to some extent, investigating whether and how they diverge is a useful way to drive a wedge between explanation and causation, potentially revealing the different roles they play in human cognition. In particular, we hypothesize that explanation plays a special role in guiding generalization, and that (in causal domains) mechanistic understanding often underwrites such guidance. This generates the prediction that explanation claims are more sensitive to the presence (vs. absence) of a plausible mechanism than are causal claims. If true, this finding would in turn constrain theorizing about the potentially unique role of explanation in cognition, a topic we take up in the general discussion.

In the remainder of the introduction we first review past work on the roles of covariation and mechanisms in causal and explanatory judgments, and then present our hypotheses and the five experiments that test them. Throughout this work, we use “covariation” to refer to the correlation between the occurrence of a candidate cause (e.g., giving birth in the First Clinic, vs. the second) and the occurrence of a particular effect (e.g., Puerperal Fever, vs. an absence of fever), where contrasts between explanatory and causal claims should be understood as holding the candidate cause and effect constant. For instance, if the causal claim is that giving birth in the First Clinic *causes* Puerperal Fever, the corresponding explanation is that mothers contract Puerperal Fever *because* they give birth in the First Clinic. Defining “mechanism” is less straightforward, with variation within and across fields (for some discussion see Lombrozo and Vasilyeva, 2017). For present purposes, we define a mechanism as a sequence of more fine-grained causal steps mediating the relationship between the cause and effect.

### Covariation and Mechanism in Causal and Explanatory Judgments

Decades of research on causal learning have pinpointed both covariation and mechanism information as relevant to causal reasoning and the evaluation of causal claims (e.g., Ahn et al., 1995; Koslowski, 1996; Park and Sloman, 2014). One ongoing debate concerns whether more statistical, covariation-based accounts or more mechanistic accounts (e.g., describing the transfer of force from a cause to an effect) capture causal judgments better (e.g., Talmy, 1988; Cheng and Novick, 1992; Newsome, 2003; Wolff, 2007; Wolff and Barbey, 2015). Yet another proposal reconciles these debates by arguing that the two types of information play different roles in assessments of type vs. token-level causation (Danks, 2005; see also White, 1990).

By comparison, there is much less empirical work on the role of these factors in explanation judgments. Nonetheless, we have reasons to expect explanations to respond both to covariation and mechanism. For example, explanations are more likely to be inferred when they are more strongly supported by probabilistic evidence (e.g., Lombrozo, 2007), and specification of mechanism figures among uncontroversial “explanatory virtues,” or characteristics that make for better explanations (Lipton, 2004). There is also evidence that explanations with mechanistic content are beneficial for learning (e.g., Keil, 2019; Kelemen, 2019), and that they are sometimes preferred over alternatives. For instance, formal explanations, which appeal to category membership but obscure relevant mechanisms (e.g., “it freezes because it’s water”), tend to receive lower ratings than more obviously mechanistic causal and functional explanations (Lombrozo and Carey, 2006; Gelman et al., 2018; Liquin and Lombrozo, 2018; Vasil et al., 2022).^1^

Following from this research, an initial question our experiments address is whether covariation and mechanism information matter for explanatory judgments, as they do for causal judgments and as one would expect based on the considerations cited above. However, the more critical (and novel) question that our experiments address is whether covariation and mechanism information matter *differentially* for explanatory and causal judgments. For example, can missing mechanism information be more detrimental to our endorsement of explanations than to our willingness to accept that the corresponding causal relationship exists? Could strength of covariation matter more for causal judgments than for assessments of corresponding explanations?

There are a few reasons to expect that the evaluation of explanation claims (e.g., “maternal mortality was higher in the First Clinic *because* it was staffed by doctors”) may be more sensitive to mechanism information than the corresponding causal claims (e.g., “the staffing by doctors *caused* the higher maternal mortality in the First Clinic”). First, this prediction arises naturally from counterfactual, or statistical approaches to causation (Lewis, 1974, 2004; Hitchcock, 2008; Menzies, 2008), where mechanism information might provide evidence relevant to assessing causation, but it is not constitutive of it. In contrast, on some prominent accounts of explanation, at least partial specification of a mechanism is necessary for explanation. This includes mechanistic accounts of explanation in philosophy of science (Machamer et al., 2000; Lipton, 2004; Bechtel and Abrahamsen, 2005), which align well with empirical psychological evidence documenting the high value of mechanistic information in everyday explanation (Ahn and Kalish, 2002; Keil, 2019; Kelemen, 2019).

Another reason to expect divergence between causal and explanatory judgments is that, while they track similar phenomena in real life, they may serve somewhat different cognitive functions. Hints for a particularly close connection between explanation and mechanism information come from accounts of explanation characterizing it as geared toward generalizing beyond the original observations being explained (e.g., Craik, 1943; Heider, 1958). For example, according to the Explanation for Export proposal (Lombrozo and Carey, 2006), one of the key functions of explanation is to support novel predictions and generalizations. If this is correct, explanatory judgments may be particularly attuned to information supporting these functions. While both strong correlations and mechanistic connections between variables support generalization, we speculate that causal and explanatory judgments are fine-tuned to information supporting different aspects, or types of generalization. In particular, causal judgments of the type we examine in this article may prioritize the *breadth* of generalization, or maximizing the sheer number of cases for which the cited relationship will support accurate predictions (this is comparable to causal strength or effect size). In contrast, explanation may be particularly geared to offering *guidance* about how to extend observations from the explained case to novel circumstances (critically, including circumstances different from those previously observed).

This distinction between *breadth* and *guidance* is introduced in Blanchard et al. (2018b), and it can be illustrated using our introductory example. If we imagine varying the strength of the correlation between birth clinic and maternal mortality, we increase breadth insofar as the relationship between birth clinic and maternal mortality successfully describes more cases within this sample, and is likely to predict more cases as well – provided that conditions do not change. But suppose we now want to generalize to new conditions: a clinic in which doctors use sterilized gloves, or where the midwives (vs. doctors) deliver in the First Clinic. Merely knowing the correlation between birth clinic and maternal mortality in the initial conditions (even if this correlation is very high) offers little *guidance* in generalizing beyond these initial conditions, since we do not know whether the clinic itself, the attending staff, or something about their properties is responsible for the observed correlation. By contrast, understanding the mechanism by which mothers in the First Clinic are more likely to have bad outcomes offers an excellent basis for generalizing beyond the initial conditions: doctors with sterilized gloves should produce good outcomes (because this eliminates contact with the cadaveric matter or germs), whereas merely swapping birthing location (without otherwise changing doctors’ routines) will not reduce rates of fever for mothers attended by doctors. In other words, mechanism knowledge can offer effective guidance in novel, previously unobserved, and/or hypothetical circumstances, in virtue of supporting inferences about which elements of the mechanism will continue or cease functioning in that circumstance, and about the implications of such deviations for the outcome. If explanation is specifically geared toward supporting such judgments, it should display heightened sensitivity to mechanism information.

Based on similar reasoning, we might expect explanation to be relatively insensitive to the strength of covariation. First, strong covariation is not necessary for explanation. For example, we might accept infection with the SARS-CoV-2 virus as an explanation for an individual’s rare inflammatory response, even if the covariation between this infection and response is quite modest. A classic example from philosophy highlights the asymmetry between covariation strength and explanation goodness: untreated syphilis seems to be a perfectly good explanation of why a person developed paresis, even if only a small percentage of people with syphilis develop paresis, such that the causal strength of the link from syphilis to paresis is weak (e.g., Salmon, 1984).

Second, covariation alone offers limited support for generalization to novel contexts (in the absence of additional assumptions about the mechanism and/or similarity of contexts), and is thus compromised as a basis for guidance. This is not to say that covariation information does nothing to promote guidance: when it goes beyond bivariate correlations and encodes multidimensional correlation matrices, it begins to capture interactions with background variables and can shed light on which aspects of the environment are invariant under relevant conditions (Liljeholm and Cheng, 2007). But in the cases we consider – which might be most representative of the initial stages of inquiry in learning about a new causal or explanatory relationship – the covariation information is insufficient to offer much guidance.

Finally, there is more general evidence that explanatory judgments may be “special” and differ in various ways from other related judgments (see Lombrozo, 2012; Lombrozo and Vasilyeva, 2017, for reviews). When people evaluate explanatory claims, they take into account such properties of explanatory hypotheses as simplicity (preferring simpler explanations even when probabilistic evidence favors complex explanations; Lombrozo, 2007; Bonawitz and Lombrozo, 2012; Pacer and Lombrozo, 2017; Vrantsidis and Lombrozo, in press), latent scope (preferring explanations that do not make unverified predictions; Khemlani et al., 2011), explanatory power [roughly tracking confirmation, (Good, 1960) while deviating from the objective posterior probability of a hypothesis; Douven and Schupbach, 2015a,b] and other explanatory “virtues” (Lipton, 2004). This illustrates that explanation judgments are influenced by a variety of considerations beyond the covariation between cause and effect.^2^

In sum, we posit that while explanatory and causal claims will both be sensitive to information about mechanisms and covariation, this sensitivity will be unequal, such that explanatory claims will depend more than matched causal claims upon the provision (vs. omission) of a mechanism, and causal claims will depend more than matched explanation claims on the strength of covariation between the relevant cause and effect. This prediction stems from the hypothesis that explanations are key to generalization, with a special emphasis on their role in supporting guidance vs. breadth.

### Past Work Comparing Judgments of Explanation and Causation

Prior research comparing explanation and causation judgments directly is limited, and the results are somewhat mixed. While some studies comparing causation and explanation claims directly find no differences (Blanchard et al., 2018a; Vasilyeva et al., 2018), there are documented differences in predictive vs. diagnostic reasoning (which draws upon causal and explanatory judgments, respectively). For example, people perform better on tasks requiring that they control for alternative causes if they involve diagnostic rather than predictive reasoning (Fernbach et al., 2010, 2011). Furthermore, diagnostic inferences are more likely to track uncertainty about the underlying causal structure (Meder et al., 2014). These findings are consistent with the general idea that explanation judgments are geared toward more “global” reasoning, going beyond the provided information and focusing on hidden mechanisms that shape observed regularities across a variety of contexts.

Judgments of causal and explanatory claims also differ in the context of evaluating causation by omission. Livengood and Machery (2007) presented participants with vignettes in which an outcome depends upon an event not occurring (e.g., a rope not breaking). In some cases, participants disagreed with a causal claim (e.g., that the rope not breaking caused some outcome), suggesting that absences are not necessarily regarded as causes (see also Beebee, 2004). However, participants more strongly endorsed the corresponding “because” claim (that the outcome occurred because the rope did not break), which the authors interpret as evidence that causal claims (at least in the case of causation by omission) are not simply conflated with corresponding causal explanations. For our purposes, these results are promising insofar as they suggest that people meaningfully differentiate “cause” and “because” claims.

### Current Experiments

We report five experiments addressing three main questions. Our first question is whether explanation judgments are sensitive to both covariation and mechanism information. As reviewed above, there are many reasons to think they are; our studies assess this directly.

Our second question is whether explanation and causal judgments are differentially sensitive to covariation and mechanism information. This is the primary question that this project tackles, to our knowledge for the first time. Comparing causal explanations to bare statements of the causal relationship they presuppose is a promising strategy for identifying what (if anything) causal explanation requires beyond this causal claim, potentially shedding light on the unique role of explanation in cognition. Our third question is whether mechanism information is, as we suggest, a particularly effective source of guidance concerning generalization. The answer to this question can shed light on *why* causal and explanatory judgments may be differentially sensitive to covariation and mechanism information.

In order to examine how explanation judgments track covariation strength and mechanism information (Question 1), as well as to evaluate the predicted double dissociation in sensitivity (Question 2), our general approach was to manipulate the strength of covariation evidence and the specification of a mechanism, and to elicit judgments about explanation “goodness” and causal strength. We examine Question 3 by assessing the impact of mechanism and covariation information on different kinds of generalization in Experiment 4. Additionally, Experiments 1a, 1b, 2, and 4 also included exploratory comparisons of causal and explanatory judgments to claims about understanding. On some accounts, understanding amounts to a grasp of causes and/or explanations (e.g., Strevens, 2008), but empirical research has not considered how judgments of understanding relate to judgments of causal strength and explanation quality.

To preview our main results, we find that judgments of causal strength tend to be more responsive to covariation than explanation or understanding judgments, while explanation judgments tend to be more sensitive to the specification of a full mechanism than are causal judgments. We also find that mechanism information provides better support for generalization to distant cases than covariation information. This suggests that explanations, in virtue of being particularly sensitive to mechanisms, may be particularly tailored to supporting broad generalization (or *guidance* to circumstances beyond the case observed), consistent with the idea that explanatory and causal judgments serve somewhat different cognitive functions.

## Experiments 1A and 1B

In Experiments 1a and 1b we examined how the strength of covariation evidence and the provision of mechanism information influence the evaluation of explanation claims, and we compared these evaluations to those for matched causal claims. For exploratory purposes, we also included matched understanding claims (described below). In both experiments, participants learned about novel relationships between pairs of factors. The factors were selected such that they would not suggest an obvious causal relationship. For example, one pair was “raising twins” and “detecting an approaching tsunami early.” Participants were then asked to evaluate either the causal strength of the relationship (e.g., do you think there exists a *causal relationship* between raising twins and detecting approaching tsunamis early?), the goodness of an explanatory claim based on the relationship (e.g., rate how good you think the following *explanation* is: Why do some coastal residents detect approaching tsunamis early? Because they are raising twins), or their sense of understanding (e.g., do you feel you *understand* the relationship between raising twins and detecting approaching tsunamis early?). We varied two aspects of the target relationships: covariation strength and information about the mechanism. Participants learned that there was no covariation, weak covariation, moderate covariation, or strong covariation (a deterministic relationship) between the two factors. Orthogonally, we varied the amount of information revealed about the possible mechanism connecting the two factors.

In Experiment 1a, the mechanism variable took one of two values: “no mechanism information” or “full mechanism information.” In the latter condition, participants received a detailed description of the mechanism connecting the two factors in question. In Experiment 1b, the mechanism variable took the value of either “no mechanism information” or “mechanism pointer.” In the latter condition, participants were told that the factors in question are related via some unspecified mechanism, without revealing details. The “mechanism pointer” was included to determine whether the specification of a full mechanism would be necessary to observe a mechanism effect, or whether it would suffice to state that *some* mechanism connects the two factors. If people suffer from an “illusion of explanatory depth” (Rozenblit and Keil, 2002) and make do with quite skeletal mechanistic understanding (Keil, 2003), one might anticipate a boost in judgments from even a mechanism sketch or placeholder, and that this would be greater for explanation judgments than for causal judgments.

### Method

#### Participants

Participants were recruited on Amazon Mechanical Turk in exchange for $1.45 (Experiment 1a: *N* = 492; 256 women, 232 men, 2 other genders, 2 preferred not to report their gender; mean age 34, age range 18–67, three participants chose not to indicate age; Experiment 1b: *N* = 480; 250 women, 226 men, 1 other gender, 3 preferred not to report their gender; mean age 35, age range 18–71). Sample sizes for these and subsequent experiments were determined based on power analyses set to detect a small interaction effect (*f* = 0.10, equivalent to *d* = 0.20) with 0.95 power (additional details in Online Supplement 1). In all experiments, participation was restricted to users with an IP address within the United States and an approval rating of at least 95% based on at least 50 previous tasks. Additional participants (N_*Exp*1*a*_ = 217; N_*Exp*1*b*_ = 198) were excluded for failing a comprehension check for covariation tables (18 and 17), failing a memory check (199 and 181), or both (27 and 27 in Experiments 1a and 1b, correspondingly); these screening tasks are described below.

#### Materials, Design, and Procedure

Participants first completed a practice session in which they were introduced to covariation tables and received two problems that tested for comprehension. For example, one problem showed a table cross-classifying objects in terms of whether they were triangles (yes vs. no) and whether they were blue (yes vs. no), and asked participants to enter the number of blue triangles, non-blue triangles, blue non-triangles, and non-blue non-triangles. Another problem showed a table cross-classifying people as tea-drinkers or not (in general), and whether they had tea this morning (yes or no). Participants were asked to match the original covariation table with numbers in each cell with one of two tables showing approximate quantities distributed across the four cells. Participants were given feedback and allowed multiple attempts to correct wrong responses before proceeding; they were also given an option to click on an “I give up on this question” button. Participants who gave up on these questions without providing the correct responses were excluded from further analysis.

Next, participants were presented with eight cause-effect pairs, selected to minimize prior beliefs about their relationship (see Supplementary Appendix A for the full list). Half of the participants were provided with a hypothetical mechanism connecting the cause and the effect. In Experiment 1a, the mechanism was a “full mechanism” in the sense that it specified the causal steps connecting the cause to the effect. In Experiment 1b, the mechanism was a “mechanism pointer”: participants received a general statement indicating that there exists some multi-step pathway connecting the cause to the effect, but the pathway was not specified. Below is sample text from one item (see Supplementary Appendix B for task wording):

A total of 160 coastal residents living in an isolated town participated in a large survey. The survey included many questions. Two of the questions asked:

a. Whether or not the person is raising twins
b. Whether or not the person detected the approaching tsunami early (the area had been hit by a weak tsunami shortly before the survey was conducted).

These two things may or may not be related.

*No mechanism:* In fact, the researchers who designed the survey did not have any particular hypotheses about their relationship.

*Full Mechanism* (Experiment 1a): When designing the survey, the researchers thought that they would be related as follows: When people raise twins, they are exposed to two very similar things side by side on a daily basis. As a result of this exposure, they become much better than other people at noticing fine differences and changes. This ability helps them detect subtle changes in the environment that indicate an approaching tsunami.

*Mechanism Pointer* (Experiment 1b): When designing the survey, the researchers thought that they would be related by a multi-step pathway connecting raising twins to an ability to detect an approaching tsunami: When people raise twins, their experience is very different from that of people who are not raising twins. This experience may affect how they process patterns and eventually lead to an enhanced ability to detect an approaching tsunami.

**FIGURE 1:**
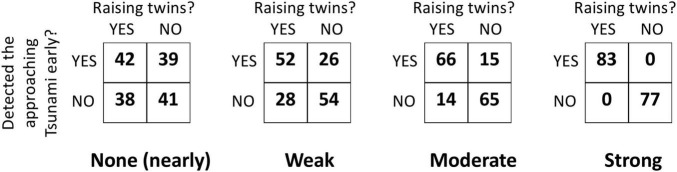
Sample covariation matrices from Experiments 1a and 1b. Conditions correspond to ΔP = 0.04, 0.33, 0.64, and 1.

Each cause-effect pair was also accompanied by a covariation table showing nearly no covariation, weak covariation, moderate covariation, or strong covariation (see Figure 1). Covariation levels rotated through cause-effect pairs across participants, and each participant saw two cause-effect pairs for each level of covariation. A small amount of noise was introduced into the covariation data in the second set of tables to avoid presenting participants with identical tables.

Participants were assigned to one of the three judgment conditions: causal strength, explanatory goodness, or sense of understanding. Judgment questions were phrased either at the type or token level. Below are sample judgments for the “twins-tsunami” item, with token wording in brackets:

[One of the respondents to the survey was AJ, who is raising twins. AJ detected the approaching tsunami early.]

Based on the information you have, …

*Causal strength:* do you think there exists a *causal relationship* between [AJ] *raising twins* and [AJ] *detecting approaching tsunamis early*? No causal relationship (1) – Very strong causal relationship (9).

*Explanatory goodness:* please rate how good you think the following *explanation* is:

Why do some coastal residents *detect approaching tsunamis early*? Because they *are raising twins*. [Why did AJ *detect the approaching tsunami early*? Because AJ *is raising twins*]. Very bad explanation (1) – Very good explanation (9).

*Sense of understanding:* do you feel you *understand* the relationship between [AJ] *raising twins* and [AJ] *detecting approaching tsunamis early*? Very weak sense of understanding (1) – Very strong sense of understanding (9).

The order of trials was randomized for each participant. Finally, as a memory check, participants sorted causes from distractors and matched them with effects; those who made one or more errors were excluded from further analyses. Participants answered demographic questions before exiting the survey.

### Results

The data from Experiments 1a and 1b were analyzed separately. Initial analyses revealed that question format (type vs. token) was not a significant predictor in either experiment: it did not significantly predict ratings in Experiment 1a or 1b (β_1*a*_ = −0.60, *p*_1*a*_ = 0.113; β_1*b*_ = 0.32, *p*_1*b*_ = 0.428) nor did it interact with other factors (Likelihood Ratios for models with and without an interaction term with question format, Experiment 1a: *LR* = 3.15, *p* = 0.925; Experiment 1b: *LR* = 2.68, *p* = 0.953). The analyses that follow therefore collapse across question format.

#### Are Explanation Ratings Sensitive to Covariation and Mechanism Information?

Explanatory goodness ratings were analyzed in a regression with covariation strength (using ΔP values calculated over the covariation data shown in the none, weak, moderate, and strong covariation conditions)^3^ and mechanism (none and strong) as predictors. We used a linear mixed-effects model fit by the maximum likelihood method, with covariation and mechanism entered as fixed effects and participant as a random effect.^4^

In Experiment 1a, both covariation and mechanism significantly predicted explanatory goodness ratings (covariation β = 3.40, *p* < 0.001, mechanism β = 0.94, *p* < 0.001; *R*^2^ = 0.22), with higher ratings the stronger the covariation (*M*_*none*_ = 3.01, *M*_*weak*_ = 4.79, *M*_*moderate*_ = 5.56, *M*_*strong*_ = 6.43) and when a full mechanism was provided (*M_*none*_* = 4.61, *M_*full*_* = 5.29).

In Experiment 1b, where full mechanism information was replaced with a mechanism pointer, covariation strength remained a significant positive predictor of explanation ratings (*M*_*none*_ = 2.44, *M*_*weak*_ = 4.43, *M*_*moderate*_ = 5.18, *M*_*strong*_ = 6.01, β = 3.53, *p* < 0.001). In contrast, the mechanism pointer did not significantly increase explanation goodness ratings, *M_*none*_* = 4.32 vs. *M_*pointer*_* = 4.73, β = 0.26, *p* = 0.233 (*R*^2^ = 0.21).

These findings suggest that explanation ratings are indeed sensitive to both covariation and mechanism information. Further, they suggest that the mechanism must be at least somewhat specified; a mere “pointer” may be insufficient.

#### Are Causation, Explanation, and Understanding Ratings Differentially Affected by Covariation Information and by Mechanism Information?

To address this question, we first ran separate mixed-effect models predicting each judgment (explanatory, causal, or understanding) from the covariation and mechanism predictors, with participant as a random effect. The resulting regression coefficients can be interpreted as reflecting the effect size for each predictor across the three judgments. To compare these coefficients across judgments, we conducted a series of permutation tests, with 999 iterations each (see Online Supplement 1 for details). We chose permutation tests as the most direct and conceptually transparent way of comparing model parameters across the three judgments, without making additional assumptions required to estimate the error variability of the relevant parameters.

**FIGURE 2:**
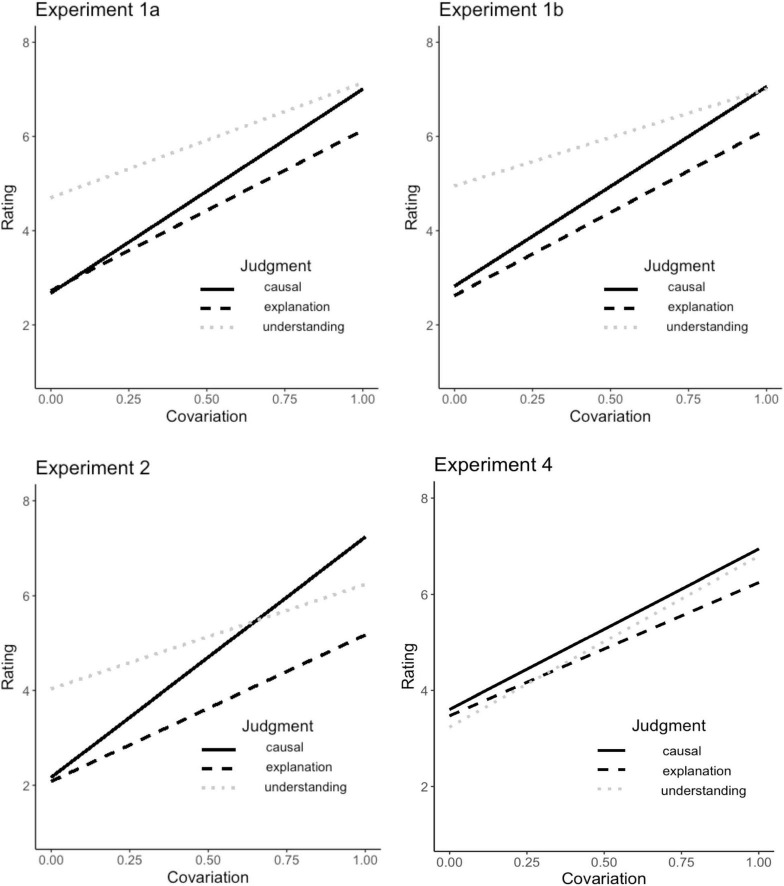
Covariation effects. Regression lines predicting ratings from covariation strength, split by judgment type: causal strength, explanatory goodness, and sense of understanding (Experiments 1a, 1b, and 2)/actual understanding (Experiment 4).

In both Experiments 1a and 1b, covariation strength positively predicted each of the three judgments (all *p*s < 0.001; see Table 1 for mean ratings). Moreover, the predictive strength of covariation varied across the three judgments: causal ratings were significantly more sensitive to covariation than were explanatory ratings (Experiment 1a: β_*caus*_ = 4.33 vs. β_*expl*_ = 3.40, *p* < 0.008; Experiment 1b: β_*caus*_ = 4.23 vs. β_*expl*_ = 3.53, *p* < 0.040) or understanding ratings (Experiment 1a: β_*unde*_ = 2.44, *p* < 0.001; Experiment 1b: β_*unde*_ = 2.06, *p* < 0.001); the latter two also differed significantly (Experiment 1a: *p* = 0.006; Experiment 1b: *p* < 0.001; see Figure 2).

**TABLE 1:** Mean ratings as a function of covariation strength and judgment in Experiments 1a, 1b, 2, and 4.

	Causal ratings	Explanatory ratings	Understanding ratings
**Experiment 1a**			
No covariation	2.43 (1.76)	3.01 (2.14)	4.68 (2.82)
Weak covariation	5.26 (2.31)	4.79 (2.29)	6.27 (2.29)
Medium covariation	5.97 (2.49)	5.56 (2.37)	6.81 (2.15)
Strong covariation	6.89 (2.80)	6.43 (2.85)	7.15 (2.48)
**Experiment 1b**			
No covariation	2.61 (1.98)	2.44 (1.78)	4.66 (2.76)
Weak covariation	4.97 (2.34)	4.43 (2.34)	5.99 (2.39)
Medium covariation	5.71 (2.57)	5.18 (2.62)	6.35 (2.37)
Strong covariation	6.93 (2.87)	6.01 (2.97)	6.77 (2.59)
**Experiment 2**			
No covariation	2.59 (1.80)	2.76 (2.01)	4.57 (2.61)
Strong covariation	7.46 (2.45)	5.74 (3.02)	6.69 (2.52)
**Experiment 4**			
No covariation	4.22 (2.20)	4.31 (2.20)	4.06 (2.17)
Strong covariation	6.54 (2.46)	6.15 (2.56)	6.49 (2.51)

*SDs shown in parentheses.*

**FIGURE 3:**
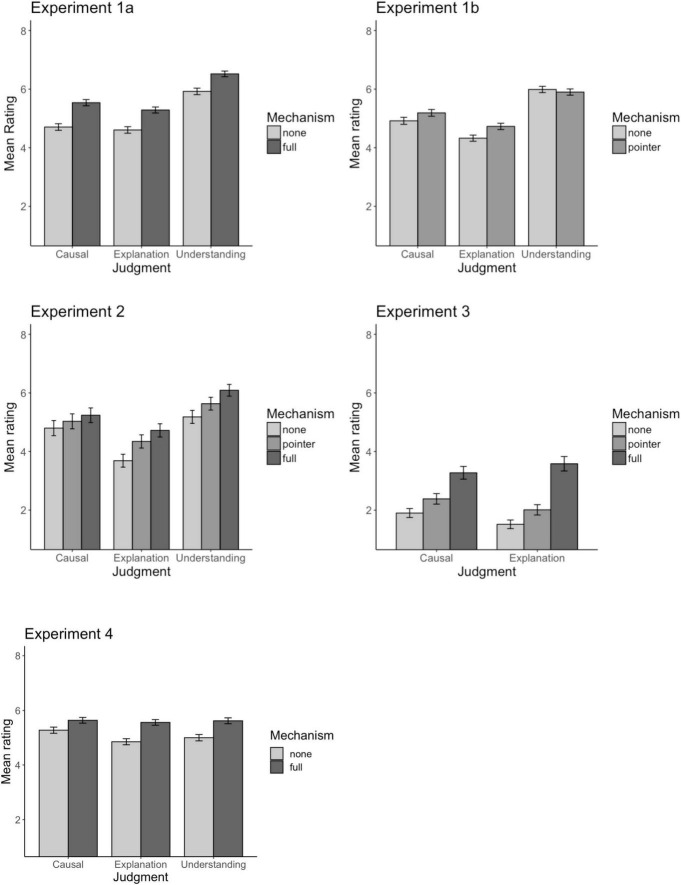
Mechanism effects. Mean ratings as a function of mechanism and judgment type: causal strength, explanatory goodness, and sense of understanding (Experiments 1–3)/actual understanding (Experiment 4). Error bars: 1SEM.

Sensitivity to mechanism information varied across Experiments 1a and 1b (see Figure 3). In Experiment 1a, the full mechanism positively predicted each of the three judgments (β_*caus*_ = 0.57, *p* = 0.002; β_*expl*_ = 0.94, *p* < 0.001; β_*unde*_ = 0.60, *p* = 0.025). Based on the regression coefficients, mechanism information appeared to be a weaker predictor of causal ratings (β_*caus*_ = 0.57) than of explanation ratings (β_*expl*_ = 0.94), but this difference did not reach significance, *p*_*Exp*1*a*_ = 0.224. Other pairwise comparisons between judgments likewise failed to reach significance (causal vs. understanding: *p*_*Exp*1*a*_ = 0.942; explanatory vs. understanding: *p*_*Exp*1*a*_ = 0.636).

In Experiment 1b, the mechanism pointer did not significantly boost any of the three target judgments (β_*caus*_ = 0.23, *p* = 0.268; β_*expl*_ = 0.26, *p* = 0.233; β_*unde*_ = −0.08, *p* = 0.784). Moreover, the differences in the predictive power of mechanism information across judgments were not significant for any pair of judgments (causal vs. explanatory: *p*_*Exp*1*b*_ = 0.904; causal vs. understanding: *p*_*Exp*1*b*_ = 0.558; explanatory vs. understanding: *p*_*Exp*1*b*_ = 0.568).

### Discussion

Experiment 1a found that explanations were judged better the stronger the corresponding covariation evidence, and when a full mechanism was provided. We also found that explanation judgments were less sensitive to covariation evidence than were causal judgments, but more sensitive than were understanding judgments. The effect of mechanism did not differ significantly across judgment types; this is a question that we revisit in Experiment 2.

Experiment 1b replicated the effect of covariation on explanatory judgments: covariation affected the three judgments to a different extent. Experiment 1b also showed that a mechanism pointer may not be sufficient to boost any of the three judgments, but this is also a question that we revisit in Experiment 2.

## Experiment 2

Although providing detailed mechanisms in Experiment 1a boosted all ratings, with a numerically higher boost for explanation judgments relative to causal judgments (as predicted), this relative difference was not statistically significant. This could indicate that our initial hypothesis was incorrect. However, it is also possible that we failed to find the predicted, differential effects of mechanism information because of the studies’ designs: Experiments 1a and 1b were presented to participants as studies about the way people understand data tables, they guided participants through an extensive practice session focusing on covariation tables, and while covariation information was manipulated within subjects (potentially drawing attention to variation in covariation), mechanism information was manipulated between subjects. To address these concerns, we conducted Experiment 2, in which we minimized task features that drew attention to the covariation tables, hoping that it would set an “even playing field” for covariation and mechanism manipulations. We also combined the mechanism manipulations from Experiments 1a and 1b into a single variable with three levels (no mechanism, mechanism pointer, and full mechanism), and we manipulated mechanism information within subjects, along with two levels of covariation (none and strong).

### Method

#### Participants

Two-hundred-and-fifty-one participants were recruited on Amazon Mechanical Turk as in Experiments 1a and 1b in exchange for $1.55 (127 women, 121 men, 1 other gender, 2 did not report their gender; mean age 34, age range 18–68). An additional 81 participants were excluded for failing a memory check for which they were asked to separate distractors from causes that had been mentioned in the study, and match causes with effects; those who made one or more errors were excluded.

#### Materials, Design, and Procedure

Mechanism information (none, pointer, and full) and covariation strength (none and strong) were manipulated within subjects, and rotated through items across participants. The type of judgment (explanation goodness, causal strength, and sense of understanding) was manipulated between subjects.

The materials and procedure were the same as in Experiments 1a and 1b, with the following exceptions: the number of items (cause-effect pairs) was reduced to 6 to accommodate the changes in the design, and the practice session was shortened. Specifically, the comprehension questions about covariation tables were removed to avoid pragmatic cues that covariation evidence should be prioritized over mechanism information during the task. Finally, because the type/token manipulation in Experiments 1a and 1b did not affect judgments, all questions were presented in the token format.

### Results

#### Are Explanation Ratings Sensitive to Covariation and Mechanism Information?

Explanatory goodness ratings were analyzed in a regression with covariation strength (as in Experiments 1a and 1b) and mechanism (none, pointer, and strong) as predictors, using a linear mixed-effects model (see Online Supplement 1 for model details). We entered the mechanism predictor as a categorical factor, and specified treatment contrasts (comparing all levels to the reference condition of “no mechanism”).

This analysis revealed that covariation and both types of mechanism information significantly predicted explanatory goodness ratings (covariation β = 3.09, *p* < 0.001, mechanism pointer β = 0.66, *p* < 0.009; full mechanism β = 1.04, *p* < 0.001; *R*^2^ = 0.27): ratings increased with covariation strength (*M*_*none*_ = 2.76, *M*_*strong*_ = 5.74) and when a pointer or full mechanism was provided (*M_*none*_* = 3.69, *M_*pointer*_* = 4.34, *M_*full*_* = 4.72).

To obtain a comparison between the pointer and full mechanism conditions, the model was re-run with “full mechanism” as a reference group. This revealed that full mechanism information did not significantly boost explanation ratings over the mechanism pointer, β = 0.38, *p* = 0.134.

#### Are Explanation, Causation, and Understanding Ratings Differentially Affected by Covariation Information and Mechanism Information?

The linear mixed-effects model for explanation ratings was repeated for causal and understanding ratings. As in Experiments 1a and 1b, we used permutation tests to run pairwise comparisons between the regression coefficients across the three judgments.

Although all judgments were positively predicted by covariation strength (all *p*’s < 0.001), they also varied in the strength of this influence: causal judgments were influenced by covariation more strongly than were explanation judgments (β_*caus*_ = 5.07 vs. β_*expl*_ = 3.09, *p* < 0.001) or understanding judgments (β_*unde*_ = 2.20, *p* < 0.001); explanation judgments were influenced marginally more strongly than understanding judgments (*p* = 0.054; see Table 1 and Figure 2).

Replicating Experiment 1a, the presence of a full mechanism (vs. no mechanism) positively predicted all three judgments (*p*_*caus*_ = 0.037; *p*_*expl*_ < 0.001; *p*_*unde*_ < 0.001). Moreover, consistent with our hypothesis, providing participants with a full mechanism (vs. no mechanism) had a larger impact on explanatory judgments than on causal judgments (β_*caus*_ = 0.44 vs. β_*expl*_ = 1.04, *p* = 0.010; see Figure 3). Understanding ratings fell in between (β_*unde*_ = 0.91, marginally different from causal judgments, *p* = 0.056, but not different from explanatory judgments, *p* = 0.660).

Providing a mechanism pointer (vs. no mechanism) positively predicted explanatory judgments and understanding judgments, but not causal judgments (β_*caus*_ = 0.23, *p* = 0.269; β_*expl*_ = 0.66, *p* = 0.009; β_*unde*_ = 0.45, *p* = 0.043). However, the differences across these judgments were not significant, *p*_*caus vs. expl*_ = 0.120, *p*_*caus vs. unde*_ = 0.384, *p*_*expl vs. unde*_ = 0.422).

Finally, comparing the full mechanism condition to the mechanism pointer condition revealed a significant difference for understanding judgments only (β_*caus*_ = 0.21, *p* = 0.323; β_*expl*_ = 0.38, *p* = 0.134; β_*unde*_ = 0.46, *p* = 0.041). However, pairs of conditions did not differ significantly from each other in the magnitude of the effect of having a full mechanism vs. a mechanism pointer (*p*_*caus vs. expl*_ = 0.468, *p*_*caus vs. unde*_ = 0.266, *p*_*expl vs. unde*_ = 0.658).

### Discussion

As in Experiment 1a, Experiment 2 found that explanation ratings were affected both by covariation and mechanism information: explanations stating that a given outcome occurred *because* of a corresponding cause seemed better to participants when they were backed up by stronger cause-effect covariation, and when participants were aware of the mechanisms connecting causes to effects. Unlike Experiment 1b, however, Experiment 2 revealed that a mechanism pointer can have a boosting effect on explanation judgments, as well.

Experiment 2 also revealed the predicted differential effects of covariation and mechanism information across explanatory and causal ratings. Causal judgments were most affected by covariation, followed by explanation judgments and (marginally lower) understanding judgments. Explanatory judgments were affected by the full mechanism information more than causal judgments were (with understanding judgments behaving similarly to explanation). Providing the mechanism pointer produced less clear results: on the one hand, it increased all three judgments equally (as reflected by the lack of significant differences across judgments); on the other hand, this increase only reached significance for explanation and understanding judgments.

## Experiment 3

Experiment 2 found that explanation ratings were more sensitive than causal ratings when it came to detailed mechanism information, whereas causal ratings were more sensitive than explanation ratings when it came to covariation. While the differential effect of covariation was also found in Experiments 1a and 1b, the effect of mechanism information was not. We therefore sought to replicate the interaction between mechanism and judgment observed in Experiment 2 before moving forward, focusing just on the causal and explanation judgments. In addition to the full mechanism information, we kept the mechanism pointer condition, since the results of Experiment 2 involving this condition were inconclusive. We also tied the mechanism more closely to each judgment by embedding the mechanism information in the body of the explanation and causation statements themselves. To examine the robustness of the observed effects, we varied both judgment type and mechanism within subjects.

### Method

#### Participants

Ninety-one participants were recruited on Amazon Mechanical Turk as in Experiments 1–2 in exchange for $1.00 (52 women, 39 men; mean age 34, age range 18–68). An additional 16 participants were excluded for failing a memory check (same as in the previous experiments).

#### Materials, Design, and Procedure

Experiment 3 included the following changes from Experiment 2. First, the mechanism information was included in the body of the explanation or causal statement (e.g., explanation with a mechanism pointer: “AJ detected the approaching tsunami early because AJ is raising twins: when people are raising twins, they are exposed to two very similar things side by side on a daily basis. As a result of this exposure, they become much better than other people at noticing fine differences and changes. This ability helps them detect subtle changes in the environment that indicate an approaching tsunami.”; see Supplementary Appendix C for sample wording). Second, the covariation variable was dropped, as was the understanding judgment. Third, both judgment type (causal strength and explanation goodness) and mechanism (none, pointer, and full) were manipulated within subjects. Judgments were blocked, with the order of blocks randomized across participants. Prior to the second block, participants were invited to “pay attention to the changed rating scale.” Mechanism levels were randomized within each judgment block. Items rotated through conditions across participants.

### Results

#### Are Explanation and Causal Ratings Sensitive to Mechanism Information?

Data from each judgment were analyzed separately using linear mixed-effect models, with the mechanism level as a fixed effect, and allowing for a random slope and intercept for each subject.

Replicating Experiment 2, both the full mechanism (β = 2.07, *p* < 0.001) and the mechanism pointer (β = 0.50, *p* = 0.017) significantly boosted explanation ratings (over no mechanism), and the full mechanism offered an additional boost over the mechanism pointer (β = 1.57, *p* < 0.001; see Figure 3 for the mean ratings).

Relative to no mechanism, causal ratings were also boosted by a full mechanism (β = 1.37, *p* < 0.001) and (in contrast to Experiment 2) by a mechanism pointer (β = 0.48, *p* < 0.025). The additional boost from full mechanism information over a mechanism pointer was also significant (β = 0.89, *p* < 0.001).

#### Are Explanation and Causal Ratings Differentially Sensitive to Mechanism Information?

To address this question, we analyzed ratings in a mixed-effects linear model entering both the mechanism level (none, pointer, and full) and judgment type (causal and explanatory) as predictors, including the interaction term.^5^ Both predictors were treatment-coded, and the mechanism predictor was releveled to conduct pairwise comparisons between mechanism levels and assess whether these effects varied across the two judgments. The model included random slopes and intercepts for participants.

As shown in Figure 3, the differences across mechanism conditions were more pronounced for explanatory judgments than for causal judgments. As in Experiment 2, this interaction was driven by the difference between the no mechanism and full mechanism conditions: the effect of providing a full mechanism (over no mechanism) was significantly stronger for explanation judgments than for causal judgments, β = 0.69, *p* = 0.030, but the effect of providing a mechanism pointer (over no mechanism) did not vary across judgments, β = 0.01, *p* = 0.971. Comparing a full mechanism to mechanism pointer, the effect was stronger for explanation than causal judgments, β = 0.68, *p* = 0.044.

Additional analyses including block order showed that it did not affect judgments, β = 0.03, *p* = 0.768, and did not interact with any of other variables (Likelihood Ratio for the models with and without the interaction term for block order 4.33, *p* = 0.503).

### Discussion

With new wording and a within-subjects manipulation of judgment type, we replicate the dissociation between causal and explanatory judgments when it comes to the role of mechanism information: adding a full mechanism boosted explanation ratings more than it boosted causal ratings. Adding a mechanism pointer boosted ratings as well, but to the same extent for both judgments.

## Experiment 4

Experiments 1a, 1b, and 2 consistently revealed that explanatory judgments are *less* sensitive to covariation information than are causal judgments, and Experiments 2–3 both found that explanatory judgments are *more* sensitive to mechanism information than are causal judgments. Experiment 4 had two aims in building upon these results. The first aim was to further examine the robustness of these effects. The second aim was to address the question of why these judgments have such specific profiles: what do we achieve by tracking covariation, and what do we achieve by tracking mechanisms?

With regards to the first aim, we already have some indication that these effects are robust to variations in wording: Experiment 3 replicated the findings from Experiment 2 with dependent measures that incorporated the mechanism in the claim under evaluation. In Experiment 4 we aimed to subject our hypothesis to an even more stringent test by reducing differences across judgments as much as possible. To this end, we made the following modifications.

First, instead of using unique rating scale anchors for each judgment (i.e., no causal relationship – very strong causal relationship; very bad explanation – very good explanation; very weak sense of understanding – very strong sense of understanding), we introduced the same rating scale for all three judgments (strongly disagree – strongly agree).

Second, in previous experiments the three judgments varied in whether they focused on the effect or on the relationship between the candidate cause and effect. Participants evaluating explanation claims were asked about the effect (e.g., why AJ detected the approaching tsunami early). By contrast, those making understanding and causal strength judgments were asked about the cause *and* the effect (e.g., the relationship between AJ having twins and AJ detecting the tsunami early). To address this, we changed the task so that all judgments involved rating agreement with statements about relationships between two events.

Third, in order to equate prior expectations that a relationship between cause and effect variables exists, we added the following phrase in both mechanism conditions: “When designing the survey, two of the three researchers involved thought these two variables might be positively correlated.” This ensured that the prior expectations about the potential relationship (its probability and direction) were equated across the no mechanism and mechanism conditions.

Finally, we modified understanding judgments to focus on actual understanding, as opposed to people’s *sense* of understanding. To do so, we had participants evaluate the value of knowing about the cause in understanding the presence of the effect (e.g., “to understand why some people detect an approaching tsunami early, it’s helpful to know that they raised twins”). This modification was made to (a) assess the generality of our findings, and (b) to eliminate another superficial difference between judgments, ensuring that all took the form of general statements about the world.

With regard to the second aim, in Experiment 4 we additionally explored two ways in which explanatory generalizations provide guidance (for relevant discussion, see Blanchard et al., 2018b; Vasilyeva et al., 2018). Specifically, we looked into the functions that covariation and mechanism could serve in supporting two types of generalization: narrow and broad.

We reasoned that covariation describes the strength of association between variables under a set of specific conditions, in a particular context, with variables taking a limited set of values. Such information is helpful in deciding whether the same relationship is likely to hold in nearly identical circumstances, e.g., deciding whether a new person who is raising twins is likely to detect an approaching tsunami. We call this type of generalization – to a different occasion, but without introducing any major changes to the variables or context – *narrow generalization*.

Mechanism information, however, may provide additional scaffolding when one needs to generalize a relationship to a set of somewhat different variables, and/or occurring under different circumstances, e.g., when one is trying to decide whether the same relationship would hold between being an expert at evaluating artwork for forgery and one’s likelihood of detecting an approaching tsunami. We call this *broad* generalization. In this case, grasping the original mechanism – that raising twins can facilitate detection of tsunamis by training the relevant skills of spotting barely detectable differences – can scaffold the inference that professional training at another activity calling for attention to fine details could likewise put one in a better position to detect an approaching tsunami (in both cases, based on the attentional mechanisms), even though on the surface raising twins and evaluating artwork for forgery do not have much in common.

To examine whether covariation and mechanism information support different kinds of generalization, Experiment 4 included measures of narrow and broad generalization.

Finally, as an additional control for participants’ attention to the provided information, we added two recall measures: covariation information recall and mechanism information recall.

### Method

#### Participants

Four-hundred-and-five participants were recruited on Amazon Mechanical Turk as in Experiments 1--3 in exchange for $3.00 (193 women, 184 men, 3 other genders; mean age 32, age range 18--74^6^). An additional 124 participants were excluded for failing a memory check (same as in the previous experiments).

#### Materials and Procedure

The materials and procedure for the *judgment task* were similar to those in Experiment 2, with the following modifications. First, the practice session was dropped; instead, participants were told to expect information about mechanisms and covariation (“sometimes you’ll read about a chain of events that could possibly connect these two variables; sometimes you’ll see how often these variables do and don’t occur together”). This further eliminated pragmatic cues that the study was primarily “about” paying attention to one type of information. Second, to equate the prior expectations regarding each cause-effect relationship, as well as the direction and valence of the potential relationship, all conditions stated that “when designing the survey, two of the three researchers involved thought these two variables might be positively correlated.” Furthermore, the relationships were introduced in the form of conditional statements, e.g., “They [the researchers] thought that if a person is raising twins, they are more likely to detect an approaching tsunami.” Third, we used different levels of covariation evidence: weak covariation corresponded to an average ΔP of 0.15 (comprised of two trials with ΔP = 0.10 and two trials with ΔP = 0.20), and strong covariation corresponded to an average ΔP of 0.85 (comprised of two trials with ΔP = 0.80 and two trials with ΔP = 0.90). The previous experiments instead used ΔP levels at/near 0 (none), 0.33 (weak), 0.66 (moderate), and 1 (strong). This modification allowed us to examine the generality of our findings to new covariation levels. Fourth, all statements presented for evaluation were about relationships between two events, mentioning both the candidate cause and effect. Fifth, the understanding judgments targeted *actual* understanding, rather than the extent to which participants felt a subjective *sense* of understanding. Finally, for all judgments the task involved rating agreement with statements using the same rating scale (strongly disagree 1 – strongly agree 9). These changes resulted in measures like the following (see Supplementary Appendix D for additional examples of stimuli):

*Causal judgment*: Raising twins has a causal influence on detecting an approaching tsunami early.

*Explanatory judgment:* Some people detect an approaching tsunami early in part because they raised twins.

*Understanding judgment*: To understand why some people detect an approaching tsunami early, it is helpful to know that they raised twins.

After the judgment task, participants completed a memory check that served as a basis for participant exclusion: as in previous experiments, participants sorted causes from distractors and matched them with effects. All participants then completed the following additional tasks: covariation table recall, mechanism recall, and generalization.

In the *covariation table recall task*, participants were provided with a blank covariation table for each cause-effect pair and asked to reproduce the data table from memory as closely as possible. The instructions indicated that “we are more interested in your general impression of how people were distributed across the four cells of the table rather than in your ability to recall exact numbers.” The entered numbers had to add to 160 (the total was visible to participants, and was automatically updated as they entered numbers).

In the *mechanism recall task*, participants were asked to write down everything they remembered about the relationship in a provided text box. For example, the instructions might say: “In Part I of the study, you were asked to consider a possible relationship between *raising twins* and *detecting an approaching tsunami early*. Please write down everything you remember from what you read about how these two factors might be related.”

These two recall tasks were included for exploratory purposes to provide insight into the mechanism by which judgment type affected ratings: we reasoned that the judgment manipulation could affect attention to different information sources, and that this could be reflected in memory.

Finally, participants completed a *generalization task*, in which they were presented with descriptions of two people, and asked to rate which person was more likely to possess the effect feature from one of the cause-effect pairs. Half of these judgments were *narrow* generalizations, and half were *broad* generations. For example, one narrow generalization read: “We’d now like you to consider two people who live in the coastal community: Tara and Susan. Tara is raising twins who are now 12 years old. Susan has never had much interaction with twins. Which of the two do you think is more likely to detect an approaching tsunami early?” Participants responded on a seven-point scale anchored at “Definitely Tara (1)” and “Definitely Susan (7),” with the midpoint labeled “equally likely.” This example illustrates narrow generalization in that one person possessed the exact cause feature familiar to participants from the judgment task.

For the broad generalization items, the target persons had a novel feature that could plausibly produce the effect using the same mechanism as the original cause feature. For example, the broad generalization item for raising twins/detecting tsunamis was that “Tara is an expert at evaluating artwork for forgery.” We reasoned that just like having twins, evaluating artwork for forgery involves attending to fine details, which, according to the proposed mechanism, could help detect subtle environmental changes preceding a tsunami. In each case, we chose broad generalization items for which we expected participants to see salient connections to the original items such that they could employ the mechanism information provided if they felt it was relevant to making the judgment. The other target person was described with an unrelated feature (e.g., Susan is a motivational speaker). Other items are described in Supplementary Appendix D.

#### Design

##### Judgment Task

Mechanism information (none and full) and covariation strength (weak and strong) were manipulated within subjects. Judgment (causal, explanatory, and understanding) was manipulated between subjects. Each participant was presented with eight cause-effect pairs in random order.

##### Covariation Table Recall Task and Mechanism Recall Task

The presentation of evidence and mechanism recall trials was blocked; the order of blocks and of trials within blocks were randomized.

##### Generalization Task

The type of generalization (narrow and broad) was manipulated within subjects and counterbalanced across items. Items were assigned to broad vs. narrow generalization such that in each condition, half of the items were presented with a full mechanism, and, orthogonally, with strong evidence in the first judgment task (assignment counterbalanced across items and participants). The order of items was randomized; the order of target persons as well as the left-right alignment of scale anchors were counterbalanced.

### Results

#### Judgment Task

##### Are Explanation Ratings Sensitive to Covariation and Mechanism Information?

Explanatory goodness ratings were analyzed in a regression with covariation strength (using ΔP values calculated for the “data tables” shown in the weak and strong covariation conditions) and mechanism information (none and full) as predictors, using a linear mixed-effects model fitted by the maximum likelihood method, with covariation and mechanism entered as fixed effects and participant as a random effect (random slopes and intercepts). Both covariation and mechanism significantly predicted explanatory goodness ratings (covariation β = 2.77, *p* < 0.001, mechanism β = 0.70, *p* < 0.001; *R*^2^ = 0.17), with higher ratings the stronger the covariation (*M*_Δ*P* = 0.10_ = 3.78, *M*_Δ*P* = 0.20_ = 4.76, *M*_Δ*P* = 0.80_ = 5.65, *M*_Δ*P* = 0.90_ = 6.65) and with explanations rated as better when a full mechanism was provided (*M_*none*_* = 4.86, *M_*full*_* = 5.56).

##### Are Explanation, Causation, and Understanding Ratings Differentially Affected by Covariation Information?

As in previous experiments, we used permutation tests to perform pairwise comparisons of regression coefficients across the three judgments.

All judgments were positively predicted by covariation strength (all *p*’s < 0.001). As shown in Figure 2, they also displayed the expected variation in the strength of this influence: causal judgments tended to be more influenced by covariation than explanation judgments, although after applying a conservative correction^7^ this difference became marginal, β_*caus*_ = 3.34 vs. β_*expl*_ = 2.77, *p* = 0.094. Understanding judgments did not differ significantly from either causal judgments or explanatory judgments (β_*unde*_ = 3.55, p_*vs. caus*_ = 0.472, p_*vs. expl*_ = 0.160). Interestingly, while all previous experiments found that understanding judgments were associated with the lowest sensitivity to covariation (though not always significantly so), in this experiment they showed a (non-significant) trend for greater sensitivity. We speculate that this could be due to the modified understanding judgments, which shifted from a focus on the subjective *sense* of understanding to a more objective form of *actual* understanding.

##### Are Explanation, Causation, and Understanding Ratings Differentially Affected by Mechanism Information?

As shown in Figure 3, providing full mechanism information positively boosted each judgment over no mechanism information, all *p*s ≤ 0.001. Consistent with Experiments 2 and 3, a full mechanism offered a stronger boost to explanatory judgments than to causal judgments (β_*caus*_ = 0.37 vs. β_*expl*_ = 0.70, *p* = 0.048). The boost for understanding ratings fell in between, and did not differ significantly from the other two judgments (β_*unde*_ = 0.60, *p*_*vs.caus*_ = 0.176, *p*_*vs.expl*_ = 0.738).

#### Evidence and Mechanism Recall Tasks

Judgment type did not have a reliable effect on accuracy for the evidence or mechanism recall tasks (see Online Supplement 2 for full analyses). This suggests that judgment type did not affect ratings by generating differential attention to different information sources, which we might have expected to affect the accuracy of later memory.

#### Generalization Task

We analyzed generalization ratings^8^ as the dependent variable in a linear mixed-effects model fit by the maximum likelihood method, with mechanism (2: none and full), covariation (2: weak and strong),^9^ generalization type (2: narrow and broad) and judgment (3: causal, explanatory, and understanding) as sum-coded categorical predictors, allowing for random participant intercepts. All these factors, with the exception of judgment type (*p* = 0.164), significantly predicted generalization ratings. Participants were more likely to generalize when the full mechanism was provided than when it was not, β = 0.59, *p* < 0.001, and when covariation was strong rather than weak, β = 0.26, *p* < 0.001. Not surprisingly, generalization ratings were also higher for narrow generalization items than for broad generalization items, β = 0.99, *p* < 0.001.

**FIGURE 4:**
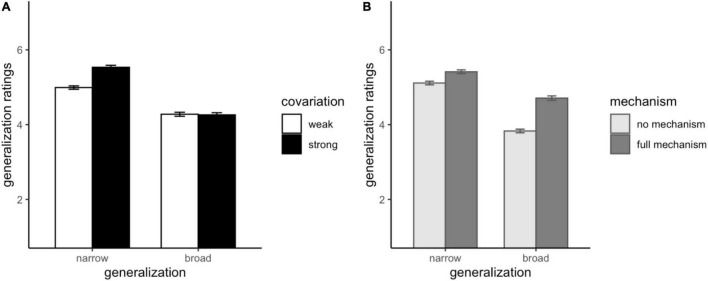
Generalization ratings as a function of generalization type and covariation strength **(A)** or as a function of generalization type and mechanism **(B)** in Experiment 4. Error bars represent 1 SEM.

Crucially, these effects were qualified by two significant two-way interactions, which we examined by switching to treatment contrasts for predictor variables and releveling the model; the judgment predictor was dropped at this point. First, as shown in Figure 4A, the manipulation of covariation strength affected narrow generalization, but not broad generalization (simple effect of covariation for narrow generalization β = 0.54, *p* < 0.001; for broad generalization β = −0.02, *p* = 0.734; interaction Likelihood Ratio = 35.23, *p* < 0.001). Second, as shown in Figure 4B, although both narrow and broad generalization received a significant boost from a mechanism, the mechanism manipulation had a significantly larger effect on broad generalization than on narrow generalization (simple effect of mechanism for narrow generalization β = 0.30, *p* < 0.001; simple effect of mechanism for broad generalization β = 0.88, *p* < 0.001; interaction Likelihood Ratio = 33.04, *p* < 0.001).

Including all the other interaction terms into a fully saturated model with a four-way interaction did not significantly improve the model fit, Likelihood Ratio = 16.98, *p* = 0.387.

### Discussion

Experiment 4 successfully replicated key effects from Experiments 1–3 under more stringent conditions. Despite matching judgments and rating scales more closely, we observed a stronger effect of covariation strength on causal ratings than on explanation ratings, and a stronger effect of mechanism information on explanation ratings than on causal ratings.

Additionally, the added recall tasks established no differences in memory for covariation and mechanism information across explanation vs. causal vs. understanding judgments, ruling out low-level accounts of observed judgment differences in terms of differential attention to these types of information.

Experiment 4 also went beyond studies 1–3 by measuring effects on generalization. Both strong covariation and mechanism information boosted narrow generalization – that is, generalization from the observed situation to a novel case involving similar circumstances. However, only mechanism information boosted broad generalization – that is, generalization to a novel situation that could plausibly involve the same mechanism. This sheds light on the functional value of mechanism information, and supports the idea that explanation – which is highly sensitive to such information – plays a special role in supporting generalization by offering guidance. In an experiment reported in the Online Supplement, we replicate these generalization findings without the preceding judgment task to ensure their reliability.

## General Discussion

Across five experiments, we report evidence that judgments of explanation quality and causal relations track different kinds of information about the world: explanation is more sensitive to mechanism than causal judgments, but causal judgments are more sensitive to covariation strength than explanation (although overall both judgments respond to both types of information). These patterns were weaker or stronger depending on methodological details (e.g., specifying a full mechanism in Experiment 1a was not as effective as in Experiments 2–4, which did not draw disproportionate attention to covariation tables); but on balance, the overall pattern of results was consistent across studies.

For exploratory purposes, we also included judgments of understanding (sense of understanding in Experiments 1–2, or actual understanding in Experiment 4). Like causal and explanatory judgments, participants’ agreement that they understood the relationship between variables was boosted both by covariation strength and mechanism information. Of the three judgment types, assessments of sense of understanding tended to be least responsive to covariation (Experiments 1–2), while the sensitivity of understanding to mechanism information generally fell in-between causal and explanatory judgments.

Overall, our results indicate that these three types of judgments – explanatory, causal, understanding – differ systematically when it comes to the role of covariation data and the effects of specifying a full mechanism, while tracking both kinds of information. We observed these dissociations even though we carefully matched the target relationships described by the explanatory, causal, and understanding claims. These dissociations caution against reducing these judgments to each other, such as characterizing causal explanations as (merely) assertions of the causal relationships they presuppose, or defining understanding as (merely) grasping causes and/or explanations (e.g., Strevens, 2008). If explanation claims could be reduced to the corresponding causal claims, we might anticipate differences in the absolute value of ratings assigned to each claim, but ratings for the different claims should have responded similarly to manipulations of covariation strength and mechanism, which is not what we observed; likewise for the understanding judgments. Our findings also have the methodological implication that assessments of learning and elicitations of judgments concerning causal relationships should not treat “cause” and “because” claims as necessarily tracking the same construct.

The unique profile of explanation in terms of relative sensitivity to mechanisms and relative insensitivity to the strength of covariation (while generally tracking both types of information) sheds light on the cognitive functions it serves. First, we have argued that an important function of explanation is to support generalization (Lombrozo and Carey, 2006). The fact that explanations track two types of relevant information – covariation and mechanism – is consistent with this idea. Second, we hypothesized that different kinds of generalization are scaffolded by different types of information: we proposed that covariation information supports narrow generalization (in virtue of indicating relationship strength), and mechanism information provides broader guidance with respect to the conditions under which a generalization might hold. The effects of covariation and mechanism on narrow and broad generalization observed in Experiment 4 are consistent with these claims. Specifically, we found that while both covariation and mechanism information support narrow generalization, mechanism information plays a unique role in supporting broad generalization judgments. Finally, tying together the evidence that good explanations are expected to provide mechanism information, and that mechanism information uniquely promotes broad generalization, we propose that *explanation is geared to support the cognitive function of generalization to novel contexts*. More speculatively, reduced sensitivity to covariation could serve this function too: a certain degree of resistance to over-fitting the data from a single sample could help achieve more reliable generalizations (and indeed, Williams et al., 2013 show that explanation encourages a search for broad patterns despite inconsistent data). Tracking covariation information, however, puts one in a good position to make generalizations to nearly identical causes and effects; in highly similar circumstances causal judgments may be more closely geared toward supporting this type of narrow generalization.

Overall, our findings suggest that explanatory goodness cannot be reduced, in any straightforward way, to judgments of causal relationships (or understanding). In addition to cautioning against characterizing one of these judgments in terms of another, our findings raise questions about the extent to which different kinds of explanatory and causal judgments could diverge. For instance, evaluating explanatory “goodness” could diverge from evaluations of explanation probability (see Vrantsidis and Lombrozo, in press), just as evaluations of causal structure diverge from those of strength (e.g., Griffiths and Tenenbaum, 2005).

Additionally, the differential sensitivity of explanation judgments to covariation and mechanism information invites questions about what does and does not count as an “explanatory virtue.” Could the strength of covariation be valuable for purely evidential reasons, while the specification of mechanism in an explanation is a genuine “virtue” in addition to having evidential import? These are some of the questions for further research.

## Conclusion

we demonstrate that judgments of causal strength, explanatory goodness and, to some extent, understanding respond differently to covariation and full mechanism information. Explanations surpass causal judgments in their sensitivity to a full mechanism, and the pattern is reversed for covariation. Our results inform our understanding of an understudied relationship: that between causal explanations and bare statements of the causal relationship they presuppose. Our results also present a challenge for proposals that characterize explanations as identifying causes, and that characterize understanding in terms of grasping causal relationships and/or explanations. More importantly, these patterns of divergence can begin to help us understand the different roles of these judgments in our cognitive lives.

## Data Availability Statement

The raw data supporting the conclusions of this article will be made available by the authors, without undue reservation.

## Ethics Statement

The studies involving human participants were reviewed and approved by the UC Berkeley CPHS. The patients/participants provided their written informed consent to participate in this study.

## Author Contributions

NV and TL conceptualized and designed the study. NV collected and analyzed the data and wrote the original draft. TL contributed extensive edits. Both authors contributed to the article and approved the submitted version.

## Conflict of Interest

The authors declare that the research was conducted in the absence of any commercial or financial relationships that could be construed as a potential conflict of interest.

## Publisher’s Note

All claims expressed in this article are solely those of the authors and do not necessarily represent those of their affiliated organizations, or those of the publisher, the editors and the reviewers. Any product that may be evaluated in this article, or claim that may be made by its manufacturer, is not guaranteed or endorsed by the publisher.

## Footnotes

1 In fact, some have argued that formal explanations are (modestly) explanatory because they are placeholders for essentialist causal mechanisms (Gelman et al., 2018) or point to underlying causes (Giffin et al., 2017), but this perspective is not universal (Prasada, 2017; Vasilyeva and Lombrozo, 2020).

2 When making causal judgments, people also attend to information that goes beyond covariation, such as temporal order and the dynamics of the putative cause and effect (Lagnado and Sloman, 2006; Rottman and Keil, 2012; Rottman et al., 2014) and asymmetries in intervention efficacy (Steyvers et al., 2003; Sloman, 2005); however, this information appears to be used to interpret the meaning of covariation in the course of causal inference, which differs from the use of explanatory virtues in the evaluation of explanatory statements.

3 Using causal power (Cheng and Novick, 1990; Cheng, 1997) instead of ΔP produces the same results in all experiments.

4 We did not include an interaction term as a predictor in any of the reported models since we had no theory-driven predictions about it. However, for exploratory purposes we checked whether the interaction between covariation and mechanism factors was significant. It was significant in Experiment 1a for causal [*LR* (Likelihood Ratio) = 5.39, *p* = 0.020] and explanatory (*LR* = 4.96, *p* = 0.026) ratings, but not for understanding ratings (*LR* = 0.01, *p* = 0.905). Compared to the no-mechanism condition, adding full mechanism information strengthened the effect of covariation on causal ratings, but weakened the effect of covariation on explanatory ratings. However, these patterns did not replicate in any of the subsequent experiments, so we are hesitant to draw strong conclusions from them.

5 We deemed this analysis more appropriate than permutation tests used in previous experiments, given the fully within-subjects design of this experiment.

6 Some participants did not indicate their age (*N* = 25) or gender (*N* = 24).

7 As we explain in the Online Supplement 1, for permutation tests we report *p*-values after applying a conservative correction. However, given the directional nature of our hypothesis, the relevant probability of obtaining the observed or more extreme difference actually corresponds only to the upper tail of the distribution generated in the permutation test, i.e., a one-tailed *p*-value of 0.047 (see Online Supplement 1 for more detail).

8 Generalization responses were not collected for 24 participants who completed the main task, passed the memory check and were included in the main dataset; thus, the dataset for the generalization analyses contains observations from 381 participants (with one missing datapoint for one trial).

9 Covariation was included in this analysis as a categorical predictor with two levels, weak (ΔPs of 0.10 or 0.20) and strong (ΔPs of 0.80 or 0.90), for the ease of interpretation and presentation; using actual ΔP levels as a continuous predictor does not change the results.
