# Supplementary material for: Explanations and Causal Judgments Are Differentially Sensitive to Covariation and Mechanism Information
Source: Front Psychol. 2022 Aug 1;13:911177. doi: 10.3389/fpsyg.2022.911177 (PMC9377274; doi:10.3389/fpsyg.2022.911177)
Supplement: Supplementary file 1 [file Data_Sheet_1.PDF]

Explanations and causal judgments are differentially sensitive to covariation and mechanism information

ONLINE SUPPLEMENT

Ny Vasil[a] & Tania Lombrozo[b]

[a]California State University East Bay

[b]Princeton University

Supplement S1. Sample sizes and Data analysis

Sample sizes. Sample sizes for Experiments 1a and 1b were determined based on power analyses conducted in G*Power, with the settings to detect a small interaction effect ($f = .1$, equivalent to $d = .20$) with .95 power in a mixed factorial design with a 3-level repeated measurement (setting correlation among measures to $r = .40$) and 6 between-subject condition cells (produced by crossing two between-subject factors with 2 and 3 levels each). This yielded a total sample size of 498 participants, or 83 participants per cell. Based on piloting, we expected an exclusion rate of approximately 30%, and recruited larger samples to accommodate that; our estimate proved fairly accurate.

For subsequent experiments, we adopted the same target of 83 participants per design cell, adjusting the total sample size depending on the specific experimental design. Due to lower exclusion rates in Experiments 3 and 4 (15-17%), the final sample sizes ended up larger than originally planned.

Linear mixed effects models. In Experiments 1a,1b, 2, 3 and 4, the linear mixed effects models were generated using the lme function in the R package nlme, with covariation and mechanism set as fixed factors and participant set as a random effect. The models used random slopes and intercepts for participants but not for items. The latter were originally included in the models with maximally rich random effects (Barr et al., 2013), but those did not converge, and item effects were dropped.

Permutation tests (Experiments 1a and 1b). Each test compared two judgment conditions, e.g., causal vs. explanatory. On each iteration, participants were randomly re-assigned to experimental conditions (without replacement, keeping the original n's), new mixed-effects models with the same predictors were fit for each "pseudo-condition," and the differences between the model coefficients were computed and recorded. Then the coefficient difference observed with the true participant assignment to experimental conditions was compared to the distribution of simulated differences. The observed difference was considered significant if fewer than 5% of the simulated differences were more extreme than the observed difference.

For example, to compare the regression coefficients of the covariation predictor across causal and explanatory judgments, participants in these two conditions were randomly re-sorted to "pseudo-causal" and "pseudo-explanatory" conditions, mixed-effect models with covariation, mechanism and their interaction as fixed factors were fit for each pseudo-condition, and the simulated difference between the regression coefficients for covariation variable in the two models was saved. After 999 iterations of this procedure, the actually observed difference between the covariation regression coefficients in the casual and explanatory conditions was compared to the distribution of simulated differences, to determine whether the true observed difference is extreme enough to occur in fewer than 5% of cases out of 1000 (999 simulations + 1 model fit on the actual data). The difference of the mechanism regression coefficients for causal and explanatory judgments was evaluated based on the same set of simulations, following the same procedure.

Given the directional nature of our hypotheses about causal vs. explanatory judgments (stronger effect of covariation on causal than explanatory judgments, and weaker effect of mechanism information on causal than explanatory judgments), when we use permutation tests

to compare the observed difference between causal vs. explanatory judgments to a simulated distribution of differences, the relevant probability of obtaining the observed or more extreme difference corresponds only to one tail of the distribution generated in the permutation test (falls within the top 2.5% of the distribution). Nevertheless, to err on the conservative side, we report observed one-tailed p-values multiplied by two.

Supplement S2: Additional analyses for Experiment 4

Evidence and mechanism recall tasks.

Based on the numbers participants entered in the four cells of the blank covariation tables, we calculated a "recalled ΔP value" for each cause-effect pair, and computed difference scores by subtracting from this value the actual ΔP value characterizing the relationship strength for that item (either .10 or .20 for weak relationships, and either .80 or .90 for strong relationships). These ΔP difference scores reflect recall accuracy for the strength of the relationship, where a score of zero indicates perfect accuracy, a negative score indicates underestimation, and a positive score indicates overestimation.

We analyzed Δp difference scores as the dependent variable in a linear mixed-effects model fit by the maximum likelihood method, with mechanism (2: none, full), covariation (2: weak, strong) and judgment (3: causal, explanatory, understanding) as sum-coded categorical predictors, allowing for random participant intercepts. Recall of covariation evidence varied as a function of the original covariation strength, β=.41, p<.001: participants overestimated the strength of weak relationships (M = .05) and underestimated the strength of strong relationships (M = -.37; both significantly different from zero, p's<.001; significance assessed using intercept-only models, separately for the weak and strong relationships). Recall of covariation evidence was also affected by mechanism detail, β=.04, p<.001: participants underestimated the strength of covariation more in the absence of mechanistic detail (M = -.18) than in the presence of it (M = -.14, both significantly below zero, p's<.001). Most importantly, the accuracy of memory for covariation evidence did not vary as a function of judgment type, p = .309.

There were no other effects: including interaction terms to a fully saturated model did not improve the model fit, Likelihood Ratio = 6.23, p = .513.

Mechanism recall task.

Participants' responses to the items for which they did receive full mechanisms were coded as containing recall of no mechanism (0), partial mechanism (1), or full mechanism (2). Two coders coded all responses (Cronbach's alpha = .84). Mean recall fell between partial and full, M=1.30, SD = .84. Mechanism recall ratings were analyzed in a linear mixed-effects model fit by the maximum likelihood method, with covariation (2: weak, strong) and judgment (3: causal, explanatory, understanding) as sum-coded categorical predictors, including an interaction term and allowing for random participant intercepts. Neither judgment type, p=.826, nor covariation strength, p=.559, influenced memory for mechanisms, and there was no interaction, p=.612.

Supplement S3: An additional experiment (replication with modifications of Experiment 4)

We have argued in the main paper that given the function of explanation to support generalization (Lombrozo & Carey, 2006), explanations should track both covariation and mechanism information, as both of these can underwrite generalizations. Experiments 1-4 reported in the main paper provide evidence for this claim. We have also argued that covariation information supports breadth, or narrow generalization “within sample” (in virtue of indicating relationship strength), and mechanism information supports guidance, or broad generalization “out of sample” (in virtue of indicating under what modifications in conditions the target relationship would continue or cease to hold). The effects of covariation and mechanism on narrow and broad generalization observed in Experiment 4 provide support for this claim. We have also observed that causal judgments tend to be more sensitive to covariation information (compared to explanatory judgments), while explanatory judgments tend to be more sensitive to mechanism information (compared to causal judgments), and we suggest that this pattern of results indicates that explanation is particularly tailored for supporting broad generalizations.

However, in Experiment 4 these effects on generalization were measured after participants already completed causal, explanatory, or understanding judgments, employing the provided covariation and/or mechanism information. To ensure the reliability of the generalization findings and rule out the possibility that they were an artifact of the preceding tasks differentially drawing attention to covariation vs. mechanism, we report an additional experiment replicating these generalization findings without the preceding judgment task.

Method

Participants

One-hundred-and-seven participants (49 women, 58 men; mean age 34, age range 18-72) were recruited on Amazon Mechanical Turk in exchange for $.70. An additional 40 participants were excluded for failing a memory check (same as in Experiment 4).

Materials, Design and Procedure

Participants read about four pairs of variables (see Appendix A) accompanied either by mechanism information or by a covariation table. For each pair of variables, they next completed either a narrow or broad generalization task, as described in Experiment 4. At no point during the experiment were they asked to make causal, explanatory, or understanding judgments.

The experiment had a 2 (type of provided information: mechanism vs. covariation) x 2 (information level: high or low) x 2 (generalization type: narrow vs. broad) mixed design, with the information level manipulated within subjects. Type of provided information (mechanism vs. covariation) and generalization type (narrow vs. broad) were manipulated between subjects. For participants assigned to the mechanism condition, the high information level corresponded to the full mechanism, and the low level corresponded to the no mechanism information; half of the items were presented with full mechanism information, and half of the items were presented with no mechanism information. For participants assigned to the covariation condition, the high and low information levels mapped on to the strong and weak covariation levels, correspondingly; for these participants, all items were accompanied by covariation tables, but for half of the items the relationship was strong (ΔP=.80), while for the remaining items the relationship was weak

(ΔP=.21). The assignment of items to conditions, the order of target characters in the vignettes, and the left-right alignment of scale anchors were counterbalanced; the order of items was randomized for each participant.

Results

All generalization ratings were recoded so that the higher values indicated preference for the target person who possessed the familiar cause feature (narrow generalization) or a related cause feature that relied on the same mechanism (broad generalization); this involved reversing the scale for half of the items.

A mixed ANOVA on generalization ratings as a function of generalization type, information type, and information level revealed a significant main effect of level: there were higher generalization ratings for a full mechanism / strong covariation, M=.85, compared to no mechanism / no covariation, M=.45, $F(1,103)=10.33$, $p=.002$, $\eta_p^2=.091$). There was also a significant main effect of generalization type, with higher ratings for narrow than for broad generalization, M_{narrow}=.99, M_{broad}=.30, $F(1,103)=34.03$, $p<.001$, $\eta_p^2=.248$). However, there was no main effect of information type (mechanism vs. covariation $F(1,103)=1.07$, $p=.303$).

Most importantly, the observed effects were qualified by a three-way interaction, $F(1,103)=6.19$, $p=.014$, $\eta_p^2=.057$. As shown in Figure S1, covariation information appeared to lend special support to narrow generalization: strong covariation marginally boosted narrow (p=.070), but not broad generalization (p=.352), over weak covariation. In contrast, mechanism information appeared to lend support to broad generalization more than to narrow generalization: full mechanism significantly boosted broad generalization, $p<.001$, but only marginally boosted narrow generalization, p=.096, compared to no mechanism information.

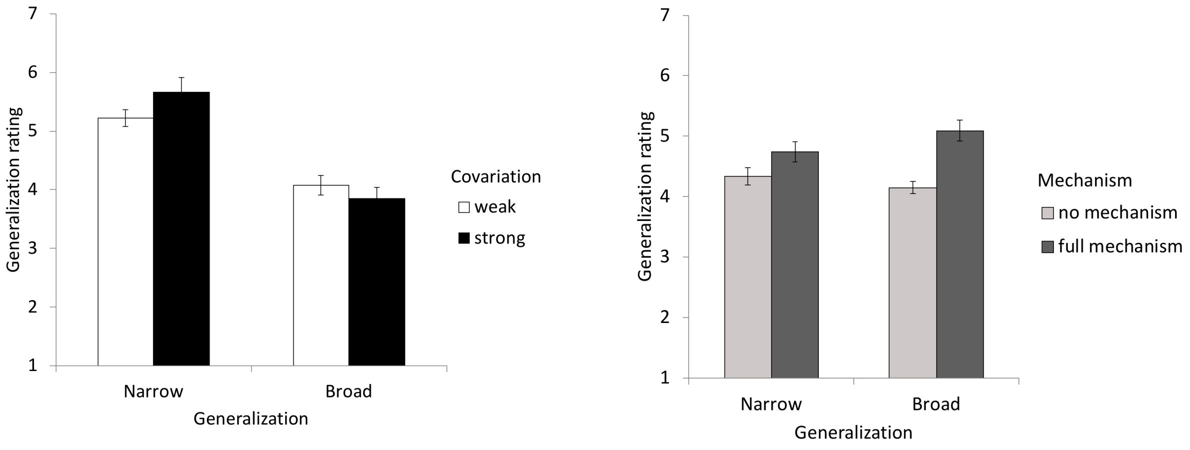

a.

b.

Figure S1. Generalization ratings as a function of generalization type and covariation strength (panel a) or as a function of generalization type and mechanism (panel b) in Experiment 5. Error bars represent 1 SEM.

References

Barr, D. J., Levy, R., Scheepers, C., & Tily, H. J.(2013). Random effects structure for confirmatory hypothesis testing: Keep it maximal. *Journal of Memory and Language*, *68*, 255-278.
